# A Character Level Based and Word Level Based Approach for Chinese-Vietnamese Machine Translation

**DOI:** 10.1155/2016/9821608

**Published:** 2016-06-29

**Authors:** Phuoc Tran, Dien Dinh, Hien T. Nguyen

**Affiliations:** ^1^Faculty of Information Technology, Ton Duc Thang University, Ho Chi Minh City 700000, Vietnam; ^2^Faculty of Information Technology, VNU-HCM University of Science, Ho Chi Minh City 700000, Vietnam

## Abstract

Chinese and Vietnamese have the same isolated language; that is, the words are not delimited by spaces. In machine translation, word segmentation is often done first when translating from Chinese or Vietnamese into different languages (typically English) and vice versa. However, it is a matter for consideration that words may or may not be segmented when translating between two languages in which spaces are not used between words, such as Chinese and Vietnamese. Since Chinese-Vietnamese is a low-resource language pair, the sparse data problem is evident in the translation system of this language pair. Therefore, while translating, whether it should be segmented or not becomes more important. In this paper, we propose a new method for translating Chinese to Vietnamese based on a combination of the advantages of character level and word level translation. In addition, a hybrid approach that combines statistics and rules is used to translate on the word level. And at the character level, a statistical translation is used. The experimental results showed that our method improved the performance of machine translation over that of character or word level translation.

## 1. Introduction

Unlike Western languages, typically English, the words in Chinese and Vietnamese are not distinguished by spaces [1]. A Chinese sentence includes a series of characters, including punctuation, and they are located next to each other without any spaces. In Vietnamese, spelling words are separated by one space, and the punctuation is located immediately after the spelling words. Therefore, the word segmentation (WS) problem often is addressed first in natural language processing in general and in machine translation (MT) from Chinese or Vietnamese into other languages in particular and vice versa.

**Figure 1:**
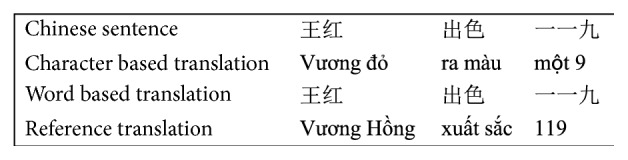
Examples for incorrect translations of CL and WL translation systems.

When translating the language pairs in which the words are not segmented by spaces, WS should be taken into consideration. Based on our experiments, we found that segmenting and not segmenting a word have their own advantages and disadvantages. The most obvious advantage of the word unsegmentation machine translation system (also called character segmentation) is that it is difficult to generate new words, but its disadvantage is that it obviously mistranslates some words. (The Chinese words in Figure 1 are some examples.) These cases are usually the translation of words in which the words have meanings that do not relate to the characters that created them, especially the Sino-Vietnamese (SINO) and named entity (NE). On the contrary, the WS machine translation system gives better translations, but it generates many unknown words (UKWs). Furthermore, in the low-resource language pairs, such as Chinese and Vietnamese, the issue of generating UKWs is worse. Figure 1 shows the errors in character level (CL) translation systems and word level (WL) translation systems in our testing corpus.

The word “王红” (“Vuong Hong”) is a Chinese person name and must be translated into Sino-Vietnamese as “Vương Hồng” [2], but in CL translation systems, the word “红” is translated into “đỏ” (red). Similarly, the word “出色” (“remarkable”) is translated into “xuất sắc” (Sino-Vietnamese word), but the CL translation systems give the translation “ra màu” (出/“ra” (“to go out,” “to come out”) và 色/“màu” (“color”)). In the remaining case, the digit string “一一九” is translated into “một 9” in the CL translation systems, while it should be “119.” The WL translation systems fail in these three cases.

Obviously, for language pairs in which the words' boundaries are not clear, such as Chinese and Vietnamese, the choice of CL or WL translation systems has its own flaws. In this paper, we propose a Chinese-Vietnamese translation model based on the combination of CL and WL translation models. At the word level, we construct the model in two STEPs.  Step 1 is to use WL statistical machine translation (WL-SMT), and Step 2 is to use rule-based MT to translate SINO and NE words, which were not translated in Step 1. In the CL model, the words that are not translated by the WL model will decay into characters and be translated based on statistics.

The rest of the paper is structured as follows: Section 2 introduces some related works.  Section 3 gives the background about Sino-Vietnamese translation and NE translation.  Section 4 provides a detailed description of our proposed model.  Section 5 shows and discusses the results of our experiments.  Section 6 summarizes our work and gives our main conclusions.

## 2. Related Work

In this paper, we focused on surveying WS methodologies to increase the performance of SMT. The term “segmented word” does not quite agree with linguists' concept of a word. A word here may be a pragmatic word (greater than the linguist's word) or a morpheme (smaller than the linguist's word). The purpose of adjusting WS is how the words in the training corpus are covered as much as possible, and the word alignment results have more 1-1 mappings.

Depending on the characteristics of translated language pairs, their WS approaches are different. To the best of our knowledge, there are some major WS approaches for MT, including morphology-based WS; anchor language-based (AL-based) WS; a combination of character segmentation and word segmentation; and other approaches.

### 2.1. Word Segmentation Based on Word Morphology

Lee [3] presented a morphological analysis algorithm for making morphologically and syntactically symmetrical language pairs with different structures. The algorithm segmented words in the rich morphological language in the form, prefix (es)- stem-suffix (es), and tagged the part of speech for bilingual corpus. Then, the algorithm detects the morphemes and decides whether to merge or delete the morphemes in the rich morphology language; the aim of this work was to make this language pair morphologically and structurally symmetrical.

In addition, in [4], Goldwater and McClosky used morphological analysis to reduce the sparse data problem in MT and increase the similarity between the two languages, thus improving the quality of machine translation for a highly inflected language, such as Czech in Czech-English translations.

### 2.2. Word Segmetation Based on the Anchor Language

In this approach, many authors based their work on one of two languages in MT to standardize WS in the other language. Chinese-English or English-Chinese translation are methods that use this approach. By using the word boundary in English, the authors conducted segment words based on the word alignments between English words and Chinese characters.

Specifically, in [5], the authors used English words as the anchor to segment the Chinese words. In this approach, the Chinese characters are combined into a word if they are aligned with the same English word. Moreover, because English NE (name of a person, name of an organization, and the name of a location) are capitalized, the Chinese words can be segmented basing on these English NEs to have better results (Chinese is case-insensitive). However, this method has a disadvantage as well as the common disadvantage in learning from bilingual corpora, which have sparse data (even though the corpora are very large) and noisy data. This leads to problems in that the system is not able to segment or it segments incorrectly the obvious words in the dictionary. Also, in this paper, the authors observed the reduction of the quality of Chinese-English machine translation when the Chinese words were not segmented.

By developing the same idea, Ma and Way [6] proposed the WS method using the results of word alignment between Chinese characters and English words. For word alignment results, the authors extracted the candidate words (*n* − 1 alignments between Chinese and English). Then, they selected the candidate words by the cooccurrence frequency of the characters inside that candidate's word.

In [7], Paul et al. expanded the WS for any language pairs for which the source language is unsegmented and the target language segmentation is determined, such as Chinese-English or Japanese-English. Furthermore, the authors proposed to decode directly from unsegmented text. They used the WS information in a phrase table to segment the input text. This avoided the inconsistency of WS between the input sentence and the phrase table.

Decomposition of Chinese words into smaller meaningful morphemes also is a popular method for improving the quality of the machine translation [8]. In this approach, first, the authors segmented the Chinese words. Then, they used the Chinese-English word alignments to filter the 1 − *n* alignments and adjust the alignments. Chinese with polysyllabic words, including more than one meaningful morpheme word, is translated into English words. For example, “教育署” is translated into “Department of Education” in English (“署” means “Department,” and “教育” means “Education”). This WS approach reduces the total cooccurrence of Chinese-English word pairs and gives more 1 − *n* alignment. For example, because “教育署” is a word, it does not contribute anything for the “教育/Education” pair and the “署/Department” pair, whereas the “教育署/Department of Education” creates the 1 − *n* alignments, such as “教育署  → Education” and “教育署  → Department.” Therefore, the authors proposed this method to decompose Chinese words into smaller, meaningful morphemes.

Similarly, Wang et al. [9] improved WS by using the results of manually aligning words from the bilingual corpus. A new point of the paper is to propose the concept of “atomic block” in the English language. The “atomic block” is the English words plus the compounds, for example, “rely on” or “carry out.” A Chinese word should be decomposed into smaller words if it aligns with any English “atomic block” (or 1 − *n* alignment).

In [10], Chu et al. segmented words for Chinese-Japanese language pairs. Both of them required WS. However, the Japanese segmenter toolkit has an accuracy up to 99%, and it is not necessary to correct WS. Japanese is used as the AL to segment Chinese. Moreover, the system also has used the shared Chinese characters (Kanji) in Japanese in order to increase the quality of Chinese WS. This work was rather close to ours, but there were some differences in the following points: (1) we translate Chinese sentences based on the combination of characters and the word level; (2) the linguistic relationships we used were NE and SINO. They are not the same as Kanji in Japanese (they are outlined in Sections 3.1 and 3.2).

According to this WS method, the unsegmented language (UL) is segmented and then, based on the word alignment results of UL and AL, the system will decay UL's words into smaller units. For example, in [8], the Chinese words that were aligned with many English words were decomposed. For example, the “洗衣机/washing machine” word pair can be decomposed into two word pairs, that is, “洗衣/washing” and “机/machine,” but the “暖气机/heater” word pair cannot be decomposed into “暖气” and “机,” because the word is only aligned with a single English word, that is, “heater.”

### 2.3. Combination of Character Segmentation and Word Segmentation

Xu et al. [11] proposed a method to perform different ways of word WS and present them as a lattice. The input sentence of the translation system is a lattice that includes different ways of WS (not a sentence in which words are segmented), and the selection of a suitable WS is performed only at the translation step. The main idea of this approach is a two-stage method, that is, from character strings to word strings and from word strings of the source language to word strings of target language.

To date, there has been only one study related to our method and that was the approach that was proposed by Zhao et al. [12]. To the best of our knowledge, this is the first approach in which Vietnamese is translated into Chinese. In their approach, they performed the translation in two steps as follows.

**Step 1 .**

Using a bilingual dictionary to find Chinese characters that correspond to Vietnamese syllables: The system performs a maximum matching algorithm on the Chinese-Vietnamese dictionary to segment the Vietnamese words. The system uses two bilingual dictionaries to segment the Vietnamese words and to convert them into Chinese characters. The first dictionary is the Sino-Vietnamese dictionary. For Sino-Vietnamese words (that appear in the dictionary), the translation process is simple, and it can be used to look up words without any ambiguities. For the words that are not Sino-Vietnamese words, the system translates them by using a phrase table. The translation system automatically collects Vietnamese monolingual corpora from the Internet. Then, these corpora are translated into Chinese by using Google Translate. After that, the system has Vietnamese-Chinese bilingual corpora. (Vietnamese is single syllables, and Chinese is characters.) Following that, the system uses GIZA++ to align words and create a phrase table. From this table, the system takes out the aligned phrases on the condition that these phrases have the same number of syllables-characters. In the case in which a Vietnamese phrase is aligned with more than one Chinese phrase, the system will choose the phrase that has the highest probability of being the correct match.

**Step 2 .**

Using monolingual Chinese to modify and change the order of words, because the order of Vietnamese words is different from the order of Chinese words. The word order must be changed to obtain the correct order in Chinese. However, Vietnamese words do not always have only one Chinese meaning. To improve this, the system replaces Chinese words (translated by Vietnamese words) by more suitable Chinese words. A synonym dictionary is used to list all possible words. The system uses a language model to change word order and to determine the most possible words to replace.

The paper also indicates some limits:

- The Sino-Vietnamese dictionary limits only two words.
- Most of the errors appear in phrase table.
- The word order after the modification is not good.

Our approach also uses Sino-Vietnamese words as a factor in the Chinese-Vietnamese translation process. However, our approach is different from the other one, as indicated below:

(i) Our approach segments Chinese words and Vietnamese words by using corpora. The main advantage of this method is that it can recognize NEs that are not covered by dictionaries.
(ii) Our phrase tables, which were created by the Computational Linguistics Center (http://www.clc.hcmus.edu.vn/) (University of Sciences, HCMC-VNU), have better quality than this work's phrase tables, which were created from the bilingual corpora collected from Google Translate.
(iii) Translation at the word level includes two stages in our system, that is, (1) translation using word level and (2) translation of SINO and NEs. In stage (1), if the words that have SINO or NE forms exist in the phrase table, they will be translated statistically. For Sino or NE forms that do not exist in the phrase table, they will be translated by rules. There are two reasons for this; that is, (1) there are some SINO words that are used less than their pure Vietnamese words, so our system will choose the meaning for the SINO words if they exist in the phrase table and (2) some foreign NEs are transliterated into Chinese (especially people's names). These kinds of NEs will have incorrect translations if they are translated by the rules of the system.
(iv) In the decoding step, we combine a statistical decoding model and a rule-based decoding model. The purpose of this strategy is to take advantage of the translation capability and word reordering capability of SMT models and the accuracy of translation by using the rules for NEs and SINO.

### 2.4. Other Methods

In this section, we present some WS methods that are different from the three methods discussed above. In [13], Zhao et al. assumed that the WS depended on the translation direction. The optimal WS is not sure to bring a better-quality translation. These authors argued that the corpus or dictionary used in WS will affect MT, so they must be optimized.

A different approach to the WS problem is based on unsupervised or semisupervised learning. In the work of Xu et al. [14], a Bayesian semisupervised learning model was proposed to segment Chinese words. They used monolingual and bilingual information to create WS suitable for SMT. Particularly, in the work of Nguyen et al. [15], an unsupervised WS model was used for MT. This model combined monolingual segmentation techniques and the bilingual word alignment model to adjust WS of the source sentence.

An extended version of this method was performed by Wang et al. [16]. The authors used the unsupervised learning method for segmenting Chinese words on large-scale corpora to support SMT. In addition, the authors modeled bilingual, unsupervised WS based on monolingual, unsupervised WS to improve the efficiency of WS, and they replaced Gibbs sampling with expectation maximization in the training process. This is considered to be the first work that used the bilingual, unsupervised WS method for large corpora. Furthermore, Zeng et al. [17] recommended using knowledge constraints to guide the monolingual supervised WS model. The authors also used “Chinese character-English word alignment” to extract word boundary distribution for character trigrams.

Like the previous approaches, these authors' methods are also applied to the language pairs in which the source language is not segmented and the word boundary of the target language is known (such as Arabic-English and Chinese-English).

## 3. Sino-Vietnamese and Named Entity

### 3.1. Sino-Vietnamese

Many Vietnamese words are borrowed from Chinese (normally called Sino-Vietnamese, which makes up about 65% of all Vietnamese words) [2]. Chinese, even in China, is pronounced differently, depending on the area, because there are many different voices or pronunciations, such as Cantonese, Hokkien, and Madarin. Some neighboring countries of China have their own reading of Chinese, such as Korea's having Sino-Korean (汉朝), Japan's having Sino-Japanese (汉和), and Vietnam's having Sino-Vietnamese (汉越). Thus, Sino-Vietnamese is the way Vietnamese people read. For example, the Chinese word “银行” is read as “yin hang” in Chinese and as “ngân hàng” in Vietnamese. A Chinese character can be pronounced as many Sino-Vietnamese words but, in a specific context, a Chinese character only corresponds to a specific Sino-Vietnamese word. As the above example “银行,” the Sino-Vietnamese word for “银” is “ngân,” and “行” is “hành,” “hạnh,” “hàng,” or “hạng”; but when “银” and “行” are combined into a word, this word is only pronounced “ngân hàng.”

Most Chinese-Vietnamese words that have Sino-Vietnamese pronunciations are Sino-Vietnamese words (called “standard Sino-Vietnamese words” or “pure Sino-Vietnamese words”). The words “văn hóa” (文化) and “hiện tại” (现在) are good examples. However, there are some Sino-Vietnamese words that have different meanings to the Vietnamese people compared to orthodox Chinese. For example, in Chinese, the word “博士” (Sino-Vietnamese is “bác sĩ” (physician in Vietnamese)) is used for doctorate and “bác sĩ” (physician in Chinese) is called “y sinh” (医生) or “đại phu” (大夫).

A Sino-Vietnamese word is the smallest meaningful unit. If we split it to smaller parts, these parts will be meaningless or have different meanings. Therefore, in Chinese-English machine translation, we do not split the Sino-Vietnamese words into smaller parts.

### 3.2. Named Entity in Chinese and Vietnamese

In this paper, we divide NEs into four categories, that is, (1) person name (PER), (2) organization name (ORG), (3) location name (LOC), and (4) number expression (NumExp) (date, time, percentage, number, and phone number). The Chinese words that belong to (1), (2), and (3) usually are translated into Vietnamese by their Sino-Vietnamese transliterations. NumExp is translated into Vietnamese by using grammatical transformation. Words in NumExp include digits combined with keywords that represent each kind of NumExp.

Like Vietnamese PER, Chinese PER are formed under the following structure: 〈family name〉〈given name〉, where both 〈family name〉 and 〈given name〉 have the length of one or two characters. For example, in the PER “赵经生” (Triệu Kinh Sinh), “赵” is a 〈family name〉 and “经生” is a 〈given name〉. In addition, to express a close relationship with elderly people, Chinese people usually use the word “老” (Lão: old) before 〈family name〉. For example, “老张” (Lão Trương) is used to refer to an elderly person who has the 〈family name〉 “Trương.” Young people are addressed in a similar manner; Chinese people usually use “小” (Tiểu: small) before 〈family name〉 to express the close relationship. For example, the word “小王” is used to call a child who has the 〈family name〉 “Vương.” This way of calling names usually omits the 〈given name〉 part in Chinese PER structures.

For Chinese LOC, the length of an LOC does not exceed 10 characters, and an LOC follows the structure 〈location name〉〈keyword〉. In this structure, 〈location name〉 is a word item in the list of Chinese location names (about 30,000 location names). It usually ends by a keyword (about 120 keywords). For example, in the word “北京市” (Beijing City), “北京” is a 〈location name〉, and “市” is a 〈keyword〉.

ORG is more complicated than person names or location names because an ORG usually includes the combination of different entities. The maximum length of an ORG is normally 15 characters. For example, in the ORG “北京语言学院” (“Học Viện Ngôn Ngữ Bắc Kinh”: “Beijing Languages Institute”), 北京 (Bắc Kinh) is an LOC, and 学院 is a 〈keyword〉.

NumExp includes some different types, such as numbers, phone numbers, order numbers, fractions, decimal numbers, dates, and times. Words in NumExp include digits combined with the keyword representing each type of NumExp. Therefore, number has an important role in constructing Chinese NumExp.

Like Vietnamese, Chinese numbers also are formed from the combination of characters that are similar to the numbers 0 to 9 in Vietnamese. The number of Chinese characters is presented in Tables 1 and 2.

A small difference between Chinese and Vietnamese is that numbers, such as 100, 1,000, 10,000, and 100,000,000, have their own words in Chinese (called unit characters). For example, the number “四百三十” is translated into “430” in Vietnamese.

## 4. Chinese-Vietnamese Translation Based on Combinations of Characters and Word Levels

### 4.1. Our Approach

We built a Chinese-Vietnamese translation model based on the combination of CL and WL translations. For the WL translation, we divided it into two phases as follows: (1) statistical translation of the WL and (2) the translation of SINO and NEs based on rule. In phase (1), some words are detected and translated into Chinese sentences. The results of this phase often are better than the results of these same words when they are translated in CL. One of hypotheses in phase (2) concerns the close relationship between the Chinese and Vietnamese languages. The statistical translation systems based on characters or words often mistranslated or did not translate NEs and SINO. Based on the relationship between the Chinese and Vietnamese languages [2], we developed some templates for the translation of NEs and SINO. The translation at the word WL often gives better results than the translation of the CL, but it generates many UKWs. Therefore, we continued to use the statistical translation system based on CL in order to translate words that could not be translated by the WL.

**Figure 2:**
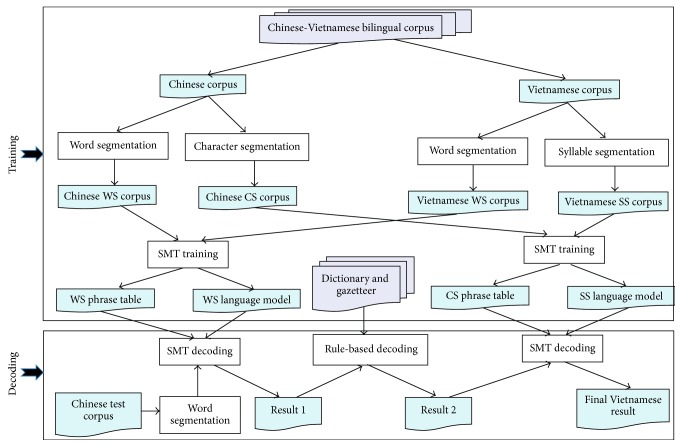
Our translation model.

Our translation system is presented in Figure 2.

### 4.2. Training Process

#### 4.2.1. Machine Translation Training Based on Statistics

The system uses SMT for both WL and CL. For WL, the Chinese corpus is segmented by the Stanford Segmenter toolkit, and the Vietnamese corpus is segmented by CLC_VN_WS of the Computational Linguistics Center (CLC). For CL, Chinese sentences have one space inserted between the characters. Similarly, one space is inserted between the spelling words and the punctuations in Vietnamese sentences. Both of the bilingual corpora are trained based on the following statistical formula [18]:

(1) v′=argmaxv⁡pv ∣ c

(2) v′=argmaxv⁡pv∗pc ∣ v,

where *v* is a Vietnamese sentence, *c* is a Chinese sentence, *v*′ is the best Vietnamese sentence, *p*(*c*∣*v*) is the translation model, and *p*(*v*) is the language model.

In this paper, we used phrase-based SMT, and formula (2) was modified as follows:

(3) v′argmaxv∏i=1Iϕci− ∣ vi−dstarti−endi−1−1·∏i=1vPLMvi ∣ v1v2⋯vi−1,

where

(4) $$\begin{array}{c} \phi \left(\;\overset{-}{c_{i}}\;|\;\overset{-}{v_{i}}\;\right)=\frac{count\left(\overset{-}{c_{i}},\overset{-}{v_{i}}\right)}{\sum_{\overset{-}{c_{i}}}^{}count\left(\overset{-}{c_{i}},\overset{-}{v_{i}}\right)}, \end{array}$$

where $\phi (\overset{-}{c_{i}}|\overset{-}{v_{i}})$ is phrase translation model, *d* is distortion model: *d*(*x*) = *α* ^|*x*|^, *α* ∈ [0,1], start_*i*_ is the position of the first word in phrase $\overset{-}{e}$, end_*i*_ is the position of the last word in phrase $\overset{-}{e}$, and *P*_LM_(*v*) is language model.

In practice, we used the Moses toolkit to train the corpora for both cases, including the word and character levels (phrase-based translation model and 3-gram language model).

#### 4.2.2. Rule-Based Machine Translation Corpora

**Figure 3:**
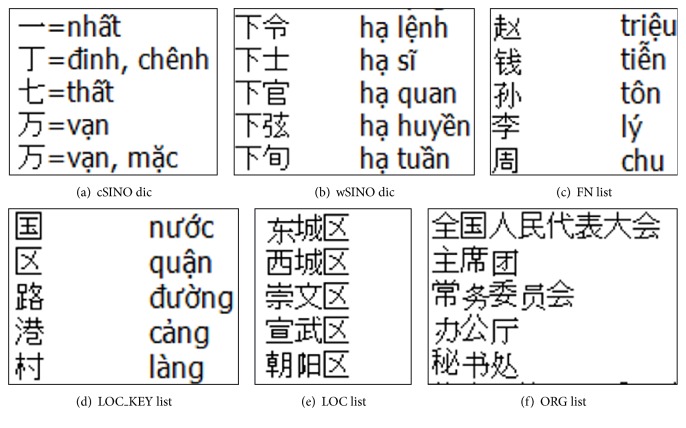
Samples of dictionaries in rule-based translation.

NEs and SINO are translated based on rule. The rule set of the system includes a CL Chinese-Sino-Vietnamese dictionary (cSINO dic), a word level Chinese-Sino-Vietnamese (wSINO), a list of Chinese family names (FN list), a keyword list of locations (LOC_KEY list), a list of the names of Chinese locations (LOC list), a list of the names of Chinese organizations (ORG list), and a set of rules for the translation of number expressions [19].  Figure 3 shows a sample of the dictionaries.

### 4.3. Decoding Phase

Our decoding phase is performed in three steps as follows.

#### 4.3.1. Decoding Based on Statistics at WL

First, the Chinese corpus is segmented at WL and translated based on SMT decoding. In practice, we used Moses decoding to translate this corpus. At this stage, we do not identify NEs and SINO in the Chinese corpus. We consider the NEs and SINO as normal words, and the translation is based completely on statistics. This is done for the following two reasons:

1. A Chinese word may have both a SINO meaning and a pure Vietnamese meaning, and the two meanings are synonyms of each other. For example, the Chinese word 现在 (now) is “hiện tại” in SINO, and its pure Vietnamese is “bây giờ,” and the two meanings are equivalent. At this stage, the system translates the word 现在 completely based on statistics.
2. The NEs in a Chinese document consist of Chinese NEs and foreign NEs. Typically, in the PER, if it is Chinese PER, the rule-based translation will give a correct result, but it will be incorrect if the PER is a foreign PER. For example, for the foreign PER, 奥巴马, the rule-based translation will give “Áo Ba Mã,” while the correct translation is “Obama.” Therefore, at this stage, we consider the NEs as normal words and translate them based on statistics.

#### 4.3.2. Decoding Based on Rules

The words in Chinese sentences are translated based on rules, including NEs (PER, LOC, ORG, and NUM) and SINO. The three types, that is, PER, NUM, and SINO, appear frequently in Chinese corpus. In this phase, the system will perform two steps sequentially, that is, NE-SINO recognition and translation.

*NE Recognition*. There are many approaches for classifying NEs, and, in this work, we divided them into four categories, that is, PER, ORG, LOC, and NUM.

First, we used the Stanford Chinese NER toolkit to identify NEs in the Chinese corpus. Then, we used a heuristic algorithm and a set of rules to filter the NEs with four labels, that is, PER, LOC, ORG, and NUM. They were adjusted as follows:

- PER: Stanford NER toolkit annotates PERSON for both Chinese PER and foreign PER. In this work, based on the Chinese FN list, we keep only the PER tag for Chinese PER and untag foreign PER.
- LOC: Stanford NER toolkit uses two tags, that is, LOC (Location) and GPE (geo-political entities), to annotate landmark, political entities. GPE indicates geographical or political names, such as cities, states, provinces, and countries. The two tags are quite close and have the same method to translate into Vietnamese, so we regroup them into a unique LOC tag.
- ORG: The Stanford NER toolkit annotates the ORG tag for the organization names.

Based on the list of the names of Chinese locations and the list of the names of Chinese organizations, the system retains only the LOC and ORG tags for Chinese location or organization entities, and it untags the foreign LOC and ORG.

- NUM: Stanford NER toolkit annotates the MISC tag (Miscellaneous) to entities related to the number, time, or a combination of other NE and non-NE components. We change from MISC tag to NUM tag for temporal and numerical entities and remove the MISC tag for the remaining entities. Specifically, the system divides the NUM tag into seven labels [19], that is, numbers that contain unit characters, numbers that do not contain unit characters, ordinal numbers, fractions, decimal numbers, date, and time. What is more, the system retains only the NUMs that have two or more digits, because NUMs that have only one digit are limited, and the statistics-based translation system can deal with it successfully.

*SINO Recognition*. Based on word level Chinese-Vietnamese dictionary and the character-level Chinese-Sino-Vietnamese dictionary, the system assigns SINO tags for the Chinese corpus and Vietnamese corpus. We annotate only SINO tags for the words that have two or more syllables. However, because of some different SINO meanings in Chinese and Vietnamese (as mentioned in Section 3.1), we only assign a SINO tag for “pure Sino-Vietnamese words.” The method is as follows.

Given that *c* is a Chinese word, *V* is a Vietnamese meaning set of* c* and* S* is a set of Sino-Vietnamese transliterations of the characters in *c*. Given *SN* = *V*∩*S*, if *SN* ≠ *ϕ*, then *c* is labeled with a SINO tag.

For example, for the Chinese word 银行, we have* V* = {ngân  hàng},* S* = {ngân hành, ngân hạnh, ngân hàng, ngân hạng} and* SN* = {ngân  hàng}; then the SINO tag will be assigned to 银行 (银行/SINO).

*Rule-Based NE and SINO Translation. *After identifying NE and SINO in a Chinese document, the system will translate these words based on rule. The translation method for each tag is presented below:

1. PER: the Chinese family name will be recognized and translated based on the “FN list,” and the Chinese given name is translated by “cSINO dic.”
2. LOC: LOC often takes the form 〈location name〉〈keyword〉. While 〈Keyword〉 is translated based on “LOC_KEY list”, 〈location name〉 is translated based on “cSINO dic.” The LOC and PER translation method is presented in detail in [20].
3. ORG: this is the most complex type of NE, because it includes NE and non-NE components. Our system uses the method in [21] to translate ORG.
4. NUM: the number expressions are translated based on the transformation rules between Chinese and Vietnamese. The rules are presented in [19].
5. SINO: Sino-Vietnamese words in Chinese sentences are translated based on the word level Chinese-Sino-Vietnamese dictionary.

#### 4.3.3. Decoding Based on Statistics at CL

In this step, the system translates the words that have yet to be translated for the first time by statistical decoding and rule-based decoding. These words include foreign NEs (PER, LOC, and ORG) and non-SINO words. They are decomposed into character CL and then translated based on statistics.

**Figure 4:**
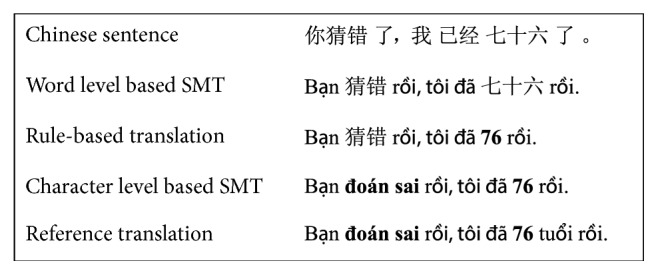
Illustration about a translation of a Chinese sentence by our system.

Figure 4 shows the translation of a Chinese sentence in the test corpus through the three phases of our system. (The English meaning of this sentence: “You guessed wrong; I am 76 years old.”)

## 5. Experiments

### 5.1. Toolkit Used in Experiments

We used the Stanford Segmenter and the Stanford NER for WS and NE recognition in Chinese, and we used CLC_VN_NER and CLC_VN_WS for WS and NE recognition in Vietnamese.

In addition, we used the GIZA++ toolkit (download at http://www.fjoch.com/giza-training-of-statistical-translation-models.html) for word or character alignment, we used the SRILM toolkit (download at http://www.speech.sri.com/projects/srilm/download.html) to train the language model, and we used the Moses toolkit (download at http://www.statmt.org/moses/?n=Moses.Releases) to train the phrase-based SMT.

### 5.2. Experimental Corpora and Evaluation Methods

The experimental bilingual corpus includes 33,372 Chinese-Vietnamese sentence pairs, which were provided by the Computational Linguistics Center (CLC) (download sample corpus: http://www.clc.hcmus.edu.vn/?page_id=32). We used 90% of the sentences for training, 5% of the sentences for testing, and the remaining 5% of the sentences for developing. The corpus of 33,372 sentence pairs was divided into three parts, that is, Part 1, which included 11,000 sentence pairs; Part 2, which had 22,000 sentence pairs; and Part 3, which was comprised of 33,372 sentence pairs. The three corpora were used to perform four experiments, that is, CL translation, WL translation, Google Translate, and translation based on our system. Tables 3, 4, and 5 show the total number of sentences, its number of words, and number of words per sentence of the experimental corpus in which, for every 20 sentences, the 1st sentence to the 18th sentence is used for training set, the 19th sentence is used for developing, and the 20th sentence is used for testing.

### 5.3. Experimental Results

We divided several sentences in the experimental corpus into five cases. The BLEU metric is the average of these five cases. For every 20 sentences in the corpus, we distributed the sentences into the corpora as shown in Table 6.

The BLEU scores and TER scores of each system are shown in Table 7. The MT output and the reference translation are segmented at the character/syllable level.

### 5.4. Analysis

The combination of statistics-based and rule-based translation at the CL and at the WL of our system aims to make use of the advantages of these methods. The advantages are include the local reordering of the phrase-based SMT, the precision of rule-based NE and SINO translation, and the capability of covering the characters of the CL translation system. The experimental results in Table 7 show that our system gives a better final translation than any of the other translation systems (as indicated by the BLEU scores and the TER scores).

For NE and SINO, the WL translation system will give the correct translation if they exist in the training corpus. Due to the limited training corpus, the WL translation system often gives many UKWs. If NE and SINO words are split into character level, the CL translation system can identify and translate them, but the results of the translation often are incorrect. For example, for the two words 王红 and 出色 in Figure 1, four characters, that is, 王, 红, 出, and 色, exist in the training corpus of the CL translation system. Their best meanings (or the highest translation probability) are “vương” (Vuong), “đỏ” (red), “ra” (to come out), and “màu” (color), respectively. In the four cases, only the 王/“Vương” pair is a correct translation. This error is due to the fact that the meaning at the CL and the meaning at the WL are not related to each other. So, the statistics-based translation at the CL for these cases is not feasible.

For these cases, the rule-based translation system will give better results. Based on the close relationship between the Chinese and Vietnamese languages, such as NE and SINO translation [2], we built the set of transformation rules in order to translate the NE and SINO, which could not translated by the WL translation system. For example, a Chinese PER 王红 must be translated into Sino-Vietnamese and must be uppercased. Its correct translation is “Vương Hồng” (not “vương đỏ” as in CL translation system). Similarly, the Sino-Vietnamese 出色 must be translated based on the Sino-Vietnamese transliteration. It means “xuất sắc” (“remarkable”), while the CL translation system gives the result as “ra màu.”

However, the rule-based translation system only can deal with words that belong to SINO or NE categories. It cannot translate all of the UKW words that cannot be translated by the WL translation system (in Step 1). In this step, the CL translation system proved to be more effective. The words that the two previous systems were unable to translate will be decomposed into characters, and, then, they were translated by the CL translation system. In this phase, the translation of the Chinese document was relatively complete. Obviously, due to the lack of resources, UKWs still may appear in the phase, but number of UKWs will not be more than that of both CL and WL translation systems.

As for Google Translate, this translation system has to translate twice when translating from Chinese into Vietnamese; the translation errors in Vietnamese side include the errors of the Chinese-English translation and English-Vietnamese translation. Moreover, Google Translate usually translates incorrectly NE words. A Chinese PER “王芳” is a good example, Google Translate transliterated it into “Wang Fang,” (Pinyin transliteration) while the correct transliteration is “Vương Phương” (Sino-Vietnamese transliteration). So, in the three corpora (11,000; 22,000; and 33,372), Google Translate has the lowest BLEU scores and gives the highest TER scores.

## 6. Conclusions and Perspectives

In this paper, we built Chinese-Vietnamese translation models by combining the strengths of two approaches, that is, statistics-based and ruled-based translation on the two levels of characters and words. This approach is suitable to the language pairs that have a low-resource requirement, have a close relationship, and a space cannot determine the word boundary. As for the language pairs, although the WL translation system often translates word more accurately than the CL translation system, it generates more UKWs. Furthermore, regarding the languages in which characters are not the smallest unit of the vocabulary, the CL translation system will incorrectly translate words with meanings that are not related to the characters that form them. Typically, in Chinese-Vietnamese MT, the named entities and Sino-Vietnamese cannot be translated properly by the CL translation system.

The experimental results indicated that our combined system significantly improved the performance of MT over the performances of both the CL and WL translation systems. The improved performance of our system is reflected in different aspects, that is, it produces fewer UKWs than the WL translation system, translates NEs and SINO better than the CL translation system, and has a higher BLEU score than either the CL translation system or the WL translation system.

Given these results, we plan to integrate more linguistic information into the system in order to increase the quality of Chinese-Vietnamese MT.

## Figures and Tables

**Table 1:** Chinese numeric characters (from 0 to 9).

Chinese numbers	零	一	二	三	四	五	六	七	八	九

Vietnamese numbers	0	1	2	3	4	5	6	7	8	9

**Table 2:** Chinese unit characters.

Chinese unit characters	十	百	千	万	亿

Vietnamese numbers	10	100	1,000	10,000	100,000,000

**Table 3:** Distribution of number of words and number of sentences in experimental corpora of 11,000 sentence pairs.

Corpora		NS	CL		WL	
			NW	NW/NS	NS	NW/NS
Chinese	Training	9,900	99,026	10.0	72,541	7.3
	Developing	550	5,645	10.3	4,138	7.5
	Testing	550	5,598	10.2	4,092	7.4

Vietnamese	Training	9,900	107,153	10.8	93,909	9.5
	Developing	550	6,151	11.2	5,401	9.8
	Testing	550	5,985	10.9	5,272	9.6

**Table 4:** Distribution of number of words and number of sentences in experimental corpora of 22,000 sentence pairs.

Corpora		NS	CL		WL	
			NW	NW/NS	NW	NW/NS
Chinese	Training	19,800	196,903	9.9	144,475	7.3
	Developing	1,100	11,292	10.3	8,237	7.5
	Testing	1,100	11,056	10.1	8,090	7.4

Vietnamese	Training	19,800	211,179	10.7	185,346	9.4
	Developing	1,100	12,028	10.9	10,534	9.6
	Testing	1,100	11,803	10.7	10,376	9.4

**Table 5:** Distribution of number of words and number of sentences in experimental corpora of 33,372 sentence pairs.

Corpora		NS	CL		WL	
			NW	NW/NS	NW	NW/NS
Chinese	Training	30,036	301,630	10.0	221,419	7.4
	Developing	1,668	16,973	10.2	12,468	7.5
	Testing	1,668	17,049	10.2	12,453	7.5

Vietnamese	Training	30,036	316,453	10.5	278,232	9.3
	Developing	1,668	17,839	10.7	15,679	9.4
	Testing	1,668	17,745	10.6	15,617	9.4

NS is “number of sentences”, NW is “number of words”, and NW/NS is “NW per NS”.

We used BLEU score and TER score to evaluate the performance of the translation systems.

**Table 6:** Distribution of number of sentences into the experimental corpora.

Case	Training corpus	Developing corpus	Testing corpus
1	From sentence 1 to sentence 18	Sentence 19	Sentence 20
2	From sentence 3 to sentence 20	Sentence 1	Sentence 2
3	From sentence 2 to sentence 19	Sentence 20	Sentence 1
4	From sentence 1 to sentence 10 and from sentence 31 to sentence 20	Sentence 11	Sentence 12
5	From sentence 1 to sentence 8 and from sentence 11 to sentence 20	Sentence 9	Sentence 10

**Table 7:** BLEU scores and TER scores of translation systems.

	CL		WL		Google translate		Our system	
	BLEU	TER	BLEU	TER	BLEU	TER	BLEU	TER
11,000	25.54	57.65	25.87	58.22	16.90	71.06	26.17	57.13
22,000	28.34	53.55	28.12	53.89	16.33	71.03	28.57	53.55
33,372	31.82	49.52	31.18	49.80	14.98	73.66	32.05	49.31
